# Nonarteritic Anterior Ischemic Optic Neuropathy following COVID-19 Vaccination: Consequence or Coincidence

**DOI:** 10.1155/2021/5126254

**Published:** 2021-10-14

**Authors:** Rika Tsukii, Yuka Kasuya, Shinji Makino

**Affiliations:** Department of Ophthalmology, Jichi Medical University, Shimotsuke, Tochigi, Japan

## Abstract

To report a patient with nonarteritic anterior ischemic optic neuropathy (NA-AION) occurring soon after the COVID-19 vaccination. A 55-year-old woman presented with a 4-day history of inferior visual field disturbance in the right eye 7 days after receiving the first dose of Pfizer-BioNTech COVID-19 vaccine. Examination revealed a best-corrected visual acuity of 20/20 in both eyes. A relative afferent pupillary defect was observed in the right eye. Fundoscopy revealed diffuse optic disc swelling in the right eye, which was prominent above the optic disc. Goldmann visual field testing identified an inferior altitudinal visual field defect with I/2 isopter in the right eye. Although typical complete inferior visual field defect was not detected, a diagnosis of NA-AION was made. The patient was followed without any treatment. During the 2-month follow-up period, the optic disc swelling was gradually improved, and visual acuity was maintained 20/20; however, the optic disc looked diffusely pale in the right eye. Although it is uncertain whether the development of NA-AION after COVID-19 vaccination was consequential or coincidental, we speculate that the close temporal relationship with COVID-19 vaccination suggests the possibility of vasculopathy on the microvascular network of optic nerve head as background of inflammatory or immune-mediated element to the timing of the onset of NA-AION. The aim of this case report is to present this biological plausibility and to elucidate potential ophthalmological complications.

## 1. Introduction

Severe acute respiratory syndrome coronavirus 2 (SARS-CoV-2) infection and the resulting coronavirus disease 2019 (COVID-19) pandemic hit the world by storm, and Japan was no exception. Currently, there has been a tremendous increase in reports of ophthalmic manifestations related with COVID-19 and its vaccination [1–3].

Nonarteritic anterior ischemic optic neuropathy (NA-AION) is an important cause of acute visual loss in middle-aged and elderly populations [4]. Typically, NA-AION is associated with risk factors such as systemic hypertension, diabetes mellitus, and optic disc morphology (small and crowded optic disc) [4].

Here, we report a case of NA-AION occurring soon after the COVID-19 vaccination.

## 2. Case Presentation

**Figure 1:**
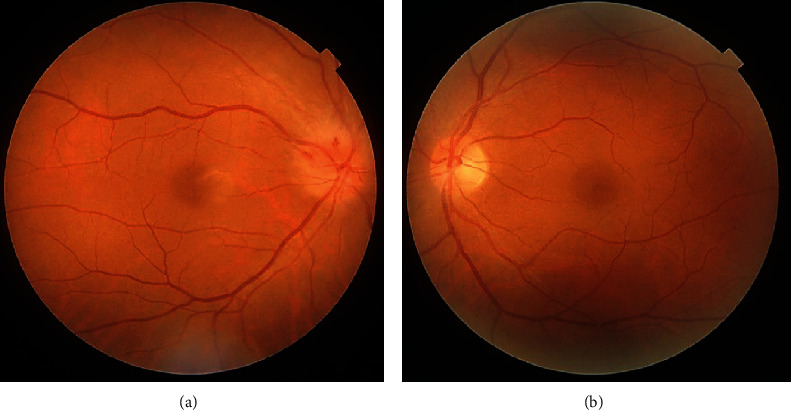
Photographs of the right (a) and left (b) fundus on initial examination. Note the diffuse optic disc swelling in the right eye, which was prominent above the optic disc.

**Figure 2:**
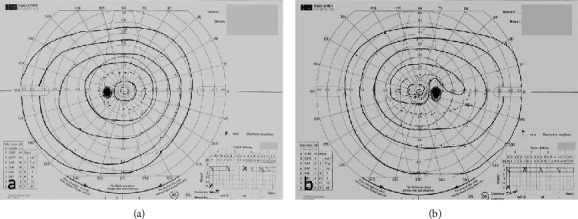
Goldmann perimetry of the left (a) and right (b) eyes. Note the inferior altitudinal visual field defect with I/2 isopter in the right eye.

A 55-year-old woman presented with a 4-day history of inferior visual field disturbance in the right eye 7 days after receiving the first dose of Pfizer-BioNTech COVID-19 vaccine (BNT162b2 mRNA COVID-19 vaccine). Her personal and family histories as well as physical examination results were unremarkable. She had no other health complaints. Examination revealed a best-corrected visual acuity of 20/20 in both eyes. A relative afferent pupillary defect was observed in the right eye. There was no abnormal ocular motility in either eye. Fundoscopy revealed diffuse optic disc swelling in the right eye, which was prominent above the optic disc (Figure 1(a)). In contrast, no abnormal findings were observed in the left eye (Figure 1(b)). Optical coherence tomography confirmed optic disc swelling in the right eye. However, no abnormal findings were observed in the right macula. Goldmann visual field testing identified an inferior altitudinal visual field defect with I/2 isopter in the right eye (Figure 2). The average critical flicker frequency value was 34 Hz in the right eye and 37 Hz in the left eye. Cranial and orbital MRI showed no abnormal findings. Laboratory examination revealed erythrocyte sedimentation rate was 11 mm/h, and C-reactive protein (CRP) level was 0.02 mg/L (reference range; 0-0.14). Myeloperoxidase-antineutrophil cytoplasmic antibody (MPO-ANCA) was 16.0 U/mL (reference range, <3.5) and proteinase 3- (PR3-) ANCA was 1.0 U/mL (reference range, <3.5). Fluorescein angiography, color vison test, cardiac echography, carotid doppler, and specialized blood coagulation examination were not available in this case.

**Figure 3:**
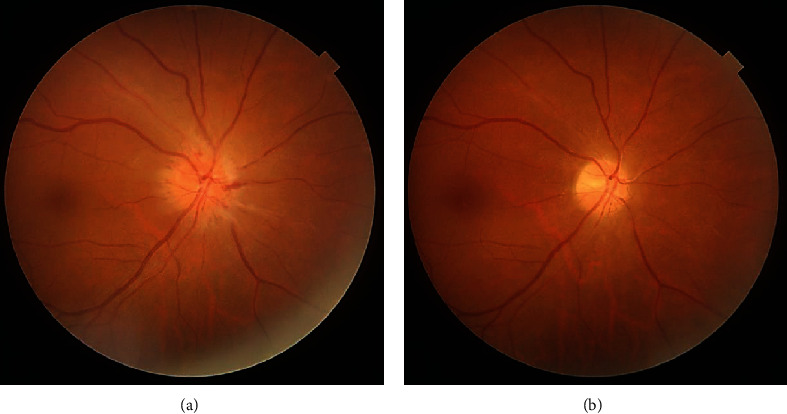
Photographs of the right fundus on 2 weeks (a) and 2 months (b) after the initial examination. Note the optic disc swelling was gradually improved; however, the optic disc looked diffusely pale.

Although typical complete inferior visual field defect was not detected nor above-mentioned examinations were not conducted, based on patient's history and the aforementioned examinations, a diagnosis of NA-AION was made. The patient was followed without any treatment. During the 2-month follow-up period, the optic disc swelling was gradually improved, and visual acuity was maintained 20/20; however, the optic disc looked diffusely pale in the right eye (Figure 3).

## 3. Discussion

NA-AION is thought to develop due to circulatory insufficiency of the posterior ciliary arteries supplying the optic nerve. Having small, crowded optic disc or other vasculopathies are risk factors for developing NA-AION. Although in the present case, the relatively young patient did not have any vasculopathic risk factors, she developed NA-AION following COVID-19 vaccination.

The occurrence and significance of autoimmune manifestations after the administration of viral vaccines remain controversial. Only two previous cases of NA-AION following influenza vaccination have been reported in the literature [5, 6]. These authors proposed an immune complex-mediated vasculopathy as a likely mechanism.

Previous cases of NA-AION in the setting of COVID-19 infection were reported in the literature [7–9]. SARS-CoV-2 can cause significant inflammation resulting in hypercoagulability manifesting as pulmonary embolism, deep-vein thrombosis, ischemic strokes, or myocardial infarcts [7]. As mentioned, patients with COVID-19 infection can manifest with hypercoagulability and hypoxemia, both of which may contribute to the development of NA-AION.

In contrast, various adverse effects have been reported with COVID-19 vaccine [2, 3], but these events' specific mechanism and frequency have not been investigated thoroughly. A previous case of arteritic anterior ischemic optic neuropathy following COVID-19 vaccination was reported [10]; however, this is the first reported case of NA-AION which was temporally related to the COVID-19 vaccination in Japan.

COVID-19 vaccines result in producing high levels of neutralizing antibodies after injection. These neutralizing antibodies recognize and target the spike proteins in the virus, killing it before the virus is disseminated and cause illness [10]. Neutralizing antibodies against SARS-CoV-2 spike proteins and/or activated T-helper-1 cells after vaccination can crossreact with proteins and antigens in large arteries, outer retinal layers, and retinal pigment epithelial cell [10]. Although it is uncertain whether the development of NA-AION after COVID-19 vaccination was consequential or coincidental, we speculate that the close temporal relationship with COVID-19 vaccination suggests the possibility of vasculopathy on the microvascular network of optic nerve head as background of inflammatory or immune-mediated element to the timing of the onset of NA-AION.

Finally, the aim of this case report is to present this biological plausibility and to elucidate potential ophthalmological complications.
